# Hydrogen-bonded co-crystal structure of benzoic acid and zwitterionic l-proline

**DOI:** 10.1107/S2056989017001785

**Published:** 2017-02-14

**Authors:** Aaron M. Chesna, Jordan M. Cox, Sanjukta Basso, Jason B. Benedict

**Affiliations:** a 764 Natural Sciences Complex, Buffalo, 14260-3000, USA; b 730 Natural Sciences Complex, Buffalo, 14260-3000, USA; c 771 Natural Sciences Complex, Buffalo, 14260-3000, USA

**Keywords:** crystal structure, co-crystal, benzoic acid, proline, hydrogen bonding

## Abstract

The title compound [systematic name: benzoic acid–pyrrolidin-1-ium-2-carboxyl­ate (1/1)], C_7_H_6_O_2_·C_5_H_9_NO_2_, is an example of the application of non-centrosymmetric co-crystallization for the growth of a crystal containing a typically centrosymmetric component in a chiral space group. It co-crystallizes in the space group *P*2_1_2_1_2_1_ and contains benzoic acid and l-proline in equal proportions. The crystal structure exhibits chains of l-proline zwitterions capped by benzoic acid mol­ecules which form a *C*(5)[*R*_3_^3^(11)] hydrogen-bonded network along [100]. The crystal structure is examined and compared to that of a similar co-crystal containing l-proline zwitterions and 4-amino­benzoic acid.

## Abstract 2

Benzoic acid–pyrrolidin-1-ium-2-carboxyl­ate (1/1) is an example of the application of non-centrosymmetric co-crystallization for the growth of a crystal containing a typically centrosymmetric component in a chiral space group. It co-crystallizes in the space group *P*2_1_2_1_2_1_ and contains benzoic acid and l-proline in equal proportions. The crystal structure exhibits chains of l-proline zwitterions capped by benzoic acid mol­ecules which form a *C*(5)[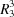
(11)] hydrogen-bonded network along [100].

## Chemical context   

Non-centrosymmetric materials are of particular importance in the field of materials chemistry for the large number of symmetry-dependent properties they can possess, including circular dichroism, pyroelectricity, and non-linear optical behavior (Halasyamani & Poeppelmeier, 1998 ▸; McMillen *et al.*, 2012 ▸; Aitken *et al.*, 2009 ▸). While purposefully engineering these materials can be difficult, one method for eliminating centrosymmetry in crystalline materials is co-crystallization with an enanti­opure chiral compound (Kwon *et al.*, 2006 ▸). In this way, provided that the chiral compound is not capable of racemization, the potential point groups are limited only to those which are chiral, and therefore non-centrosymmetric. The amino acid proline plays an important role in determining the structure of proteins, due to its structural rigidity. Proline has also been shown to be a good candidate for synthesizing non-centrosymmetric co-crystals. In fact, Timofeeva *et al.* (2003 ▸) reported success co-crystallizing di­cyano­vinyl aromatic compounds with l-proline while the same compounds would grow neat crystals when co-crystallization with l-tartaric acid was attempted.
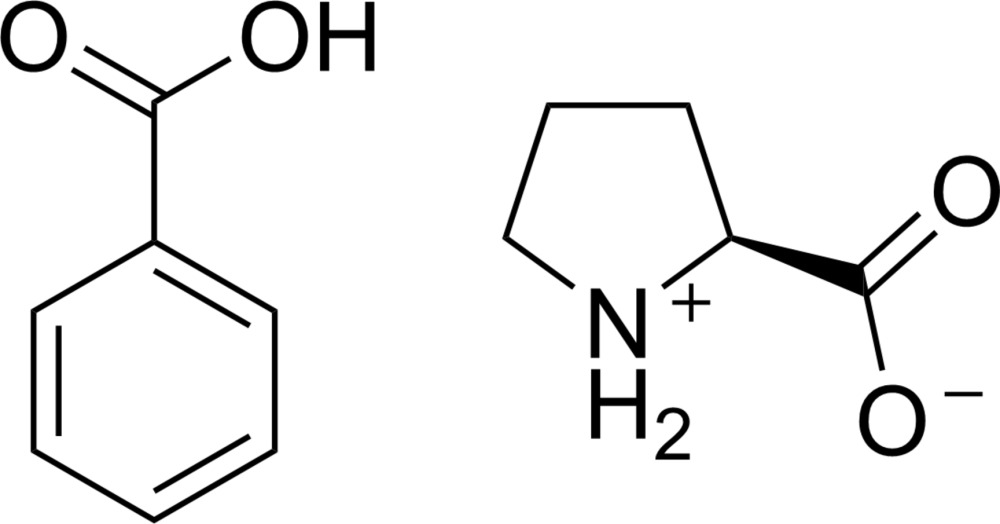


## Structural commentary   

**Figure 1:**
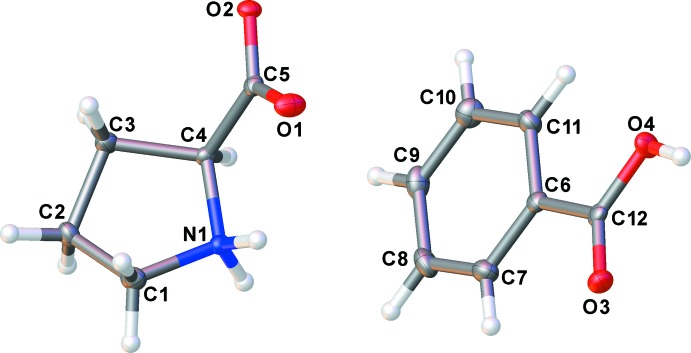
The asymmetric unit of the title compound, showing the atom-naming scheme. Displacement ellipsoids are shown at the 50% probability level.

l-proline zwitterion (**LP**) and benzoic acid (**BA**) co-crystallize in the chiral space group *P*2_1_2_1_2_1_ with one mol­ecule of l-proline and one mol­ecule of benzoic acid in the asymmetric unit, shown in Fig. 1 ▸. The l-proline exists in its zwitterionic form within the lattice while the carb­oxy­lic acid group of the benzoic acid mol­ecules remain protonated. Although the Flack parameter could not be used to unambiguously assign the absolute configuration, the enanti­omer was reliably assigned by reference to an unchanging chiral centre in the synthetic procedure.

## Supra­molecular features   

**Figure 2:**
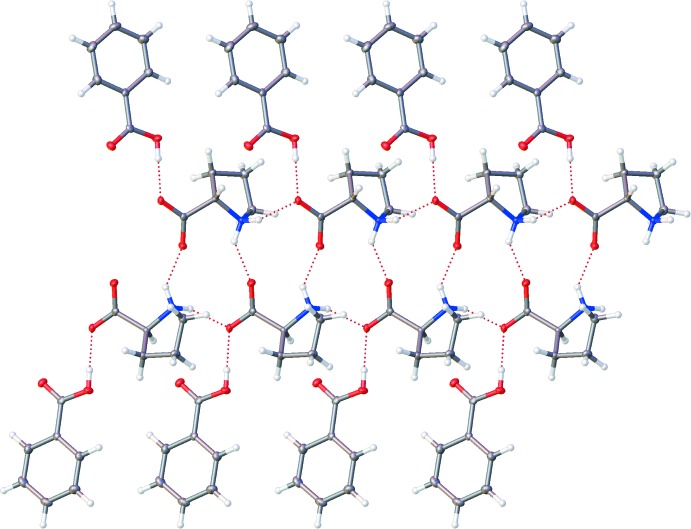
Diagram illustrating the hydrogen-bonding inter­actions in **BA–LP** co-crystal.

In this structure, each **LP** hydrogen bonds with four other **LP** mol­ecules and one **BA**. The **LP** hydrogen bonding forms 1D chains along [100] *via* (carboxyl­ate) O⋯H—N (pyrollium) inter­actions in a *C*(5)[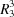
(11)] motif (Table 1 ▸). The **BA** mol­ecules act as capping groups and hydrogen bond to each of the **LP** carboxyl­ates through O—H⋯O (carboxyl­ate) inter­actions. The complete **BA–LP** chains, as shown in Fig. 2 ▸, propagate along [100] and are approximately contained in (021) and (0
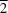
1). These chains are held together by edge–face-type π–π stacking between adjacent **BA** mol­ecules approximately along [010], with a ring-centroid to ring-centroid distance of 4.8451 (16) Å.

## Database survey   

**Figure 3:**
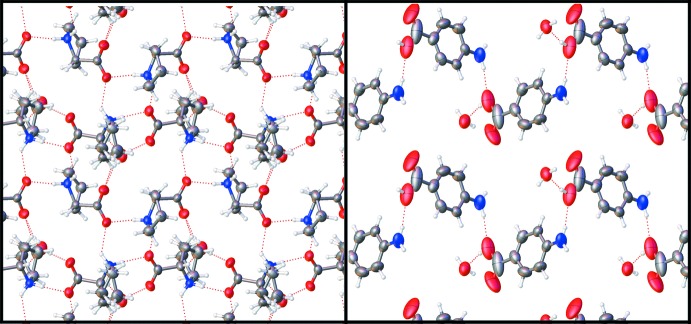
Diagram illustrating the hydrogen bonding network of **LP** in the previously reported **PABA–L**P co-crystal (left) and view of the **PABA** hydrogen-bonding network in the previously reported co-crystal (right).

Recently, the co-crystal structure of **LP** and *para*-amino­benzoic acid (**PABA**) was reported (Athimoolam & Natarajan, 2007 ▸). While the structure of **BA**–**LP** retains some structural similarities with the **PABA**–**LP** co-crystal, due to the absence of one hydrogen-bonding moiety, the amino group, the structure of **BA**–**LP** (Fig. 3 ▸) also exhibits some important differences when compared to that of **PABA**–**LP**. The head-to-tail **LP** chains in **PABA**–**LP** are similar to those in **BA**–**LP**, though instead of two chains hydrogen-bonded together to form rings, the chains hydrogen bond to form a continuous 2D sheet of **LP** mol­ecules. Much like **BA**–**LP**, the **PABA** mol­ecules hydrogen bond to the periphery of the **LP** chains; however, this crystal incorporated water into the lattice and it is to these water mol­ecules that the **PABA** mol­ecules are bound. The major difference between the two structures is the presence of the hydrogen-bond donating group at the 4-position of the **PABA** mol­ecules. This moiety allows the **PABA** mol­ecules to bridge the **LP** chains in **PABA-**-**LP**, a supra­molecular feature absent in the title compound. The result of the lack of *para*-substitution and water in the lattice is that **BA**–**LP** forms a hydrogen-bonding network which extends in only one dimension, instead of the three-dimensional network of **PABA**–**LP**.

## Synthesis and crystallization   

Solid **BA** (10.1 mg, 9.01 × 10 ^−2^ mmol) and **LP** (9.3 mg, 8.08 × 10^−2^ mmol) were added to a 25 ml scintillation vial. To this was added approximately 8 ml of ethanol followed by sonication until all solutes were fully dissolved. The loosely capped vial was then placed on an open shelf. After three weeks, colorless needle-shaped crystals of the title compound suitable for single-crystal X-ray diffraction measurements were obtained.

## Refinement   

The crystal, data collection, and refinement details are listed in Table 2 ▸. The positions of the carboxyl­ate and pyrollium hydrogen atoms were determined from the Fourier difference map, and all other hydrogen atoms were placed in idealized positions with C—H bond lengths set to 0.93 and 0.97 Å for aryl and alkyl hydrogen atoms, respectively. These hydrogen atoms were refined using a riding model with *U*_iso_(H) = 1.5*U*_eq_(O) for the carb­oxy­lic acid proton on the BA mol­ecules and *U*_iso_(H) = 1.2*U*_eq_ in all other cases. No other constraints were applied to the refinement model.

## Supplementary Material

Crystal structure: contains datablock(s) I. DOI: 10.1107/S2056989017001785/lh5828sup1.cif

Structure factors: contains datablock(s) I. DOI: 10.1107/S2056989017001785/lh5828Isup2.hkl

Supporting information file. DOI: 10.1107/S2056989017001785/lh5828Isup3.cml

CCDC reference: 1530619

Additional supporting information:  crystallographic information; 3D view; checkCIF report

## Figures and Tables

**Table 1:** Hydrogen-bond geometry (Å, °)

*D*—H⋯*A*	*D*—H	H⋯*A*	*D*⋯*A*	*D*—H⋯*A*
N1—H1*A*⋯O2^i^	0.91	1.92	2.751 (3)	151
N1—H1*B*⋯O1	0.91	2.18	2.679 (3)	114
N1—H1*B*⋯O1^ii^	0.91	2.08	2.782 (2)	133
C4—H4⋯O3^iii^	1.00	2.30	3.192 (3)	147
O4—H4*A*⋯O2^iv^	0.84	1.76	2.595 (2)	173

Symmetry codes: (i) 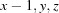; (ii) 
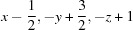
; (iii) 
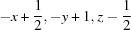
; (iv) 
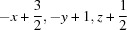
.

**Table 2:** Experimental details

Crystal data	
Chemical formula	C_5_H_9_NO_2_·C_7_H_6_O_2_
*M*_r_	237.25
Crystal system, space group	Orthorhombic, *P*2_1_2_1_2_1_
Temperature (K)	90
*a*, *b*, *c* (Å)	5.6993 (7), 12.0762 (13), 16.6839 (19)
*V* (Å^3^)	1148.3 (2)
*Z*	4
Radiation type	Mo *K*α
μ (mm^−1^)	0.10
Crystal size (mm)	0.1 × 0.01 × 0.01

Data collection	
Diffractometer	Bruker SMART APEXII area detector
Absorption correction	Multi-scan (*SADABS*; Bruker, 2013 ▸)
*T*_min_, *T*_max_	0.619, 0.746
No. of measured, independent and observed [*I* > 2σ(*I*)] reflections	13711, 2880, 2375
*R*_int_	0.069
(sin θ/λ)_max_ (Å^−1^)	0.670

Refinement	
*R*[*F* ^2^ > 2σ(*F* ^2^)], *wR*(*F* ^2^), *S*	0.043, 0.086, 1.06
No. of reflections	2880
No. of parameters	155
H-atom treatment	H-atom parameters constrained
Δρ_max_, Δρ_min_ (e Å^−3^)	0.20, −0.18
Absolute structure	Flack *x* determined using 824 quotients [(*I* ^+^)−(*I* ^−^)]/[(*I* ^+^)+(*I* ^−^)] (Parsons *et al.*, 2013 ▸)
Absolute structure parameter	0.5 (8)

Computer programs: *APEX2* and *SAINT* (Bruker, 2013 ▸), *olex2.solve* (Bourhis *et al.*, 2015 ▸), *SHELXL2014* (Sheldrick, 2015 ▸) and *OLEX2* (Dolomanov *et al.*, 2009 ▸).

## supplementary crystallographic information

### Crystal data

**Table:**

C_5_H_9_NO_2_·C_7_H_6_O_2_	*D*_x_ = 1.372 Mg m^−^^3^
*M**_r_* = 237.25	Mo *K*α radiation, λ = 0.71073 Å
Orthorhombic, *P*2_1_2_1_2_1_	Cell parameters from 2011 reflections
*a* = 5.6993 (7) Å	θ = 3.4–24.7°
*b* = 12.0762 (13) Å	µ = 0.10 mm^−^^1^
*c* = 16.6839 (19) Å	*T* = 90 K
*V* = 1148.3 (2) Å^3^	Needle, colourless
*Z* = 4	0.1 × 0.01 × 0.01 mm
*F*(000) = 504	

### Data collection

**Table:**

Bruker SMART APEXII area detector diffractometer	2880 independent reflections
Radiation source: microfocus rotating anode, Incoatec Iµs	2375 reflections with *I* > 2σ(*I*)
Mirror optics monochromator	*R*_int_ = 0.069
Detector resolution: 7.9 pixels mm^-1^	θ_max_ = 28.4°, θ_min_ = 2.1°
ω and φ scans	*h* = −7→7
Absorption correction: multi-scan (SADABS; Bruker, 2013)	*k* = −15→16
*T*_min_ = 0.619, *T*_max_ = 0.746	*l* = −22→22
13711 measured reflections	

### Refinement

**Table:**

Refinement on *F*^2^	Hydrogen site location: inferred from neighbouring sites
Least-squares matrix: full	H-atom parameters constrained
*R*[*F*^2^ > 2σ(*F*^2^)] = 0.043	*w* = 1/[σ^2^(*F*_o_^2^) + (0.0344*P*)^2^ + 0.0827*P*] where *P* = (*F*_o_^2^ + 2*F*_c_^2^)/3
*wR*(*F*^2^) = 0.086	(Δ/σ)_max_ < 0.001
*S* = 1.06	Δρ_max_ = 0.20 e Å^−^^3^
2880 reflections	Δρ_min_ = −0.18 e Å^−^^3^
155 parameters	Absolute structure: Flack *x* determined using 824 quotients [(*I*^+^)-(*I*^-^)]/[(*I*^+^)+(*I*^-^)] (Parsons *et al.*, 2013)
0 restraints	Absolute structure parameter: 0.5 (8)
Primary atom site location: iterative	

### Special details

**Table:**

Geometry. All esds (except the esd in the dihedral angle between two l.s. planes) are estimated using the full covariance matrix. The cell esds are taken into account individually in the estimation of esds in distances, angles and torsion angles; correlations between esds in cell parameters are only used when they are defined by crystal symmetry. An approximate (isotropic) treatment of cell esds is used for estimating esds involving l.s. planes.
Refinement. 1. Fixed Uiso At 1.2 times of: All C(H) groups, All C(H,H) groups, All N(H,H) groups At 1.5 times of: All O(H) groups 2.a Ternary CH refined with riding coordinates: C4(H4) 2.b Secondary CH2 refined with riding coordinates: N1(H1A,H1B), C1(H1C,H1D), C2(H2A,H2B), C3(H3A,H3B) 2.c Aromatic/amide H refined with riding coordinates: C7(H7), C8(H8), C9(H9), C10(H10), C11(H11) 2.d Idealised tetrahedral OH refined as rotating group: O4(H4A)

### Fractional atomic coordinates and isotropic or equivalent isotropic displacement parameters (Å^2^)

**Table:**

	*x*	*y*	*z*	*U*_iso_*/*U*_eq_	
O1	0.6846 (3)	0.69609 (14)	0.45367 (10)	0.0186 (4)	
O2	0.8438 (3)	0.62955 (13)	0.34146 (9)	0.0131 (4)	
N1	0.2560 (3)	0.72586 (15)	0.39137 (11)	0.0118 (4)	
H1A	0.1317	0.6787	0.3892	0.014*	
H1B	0.3091	0.7281	0.4428	0.014*	
C1	0.1825 (5)	0.8393 (2)	0.36504 (13)	0.0161 (5)	
H1C	0.2726	0.8972	0.3936	0.019*	
H1D	0.0129	0.8512	0.3745	0.019*	
C2	0.2381 (4)	0.8397 (2)	0.27615 (13)	0.0161 (5)	
H2A	0.2546	0.9162	0.2556	0.019*	
H2B	0.1152	0.8008	0.2451	0.019*	
C3	0.4714 (4)	0.7775 (2)	0.27268 (14)	0.0149 (5)	
H3A	0.6039	0.8275	0.2854	0.018*	
H3B	0.4968	0.7451	0.2189	0.018*	
C4	0.4481 (4)	0.68629 (19)	0.33630 (13)	0.0110 (5)	
H4	0.3995	0.6154	0.3101	0.013*	
C5	0.6756 (4)	0.66858 (18)	0.38222 (14)	0.0119 (5)	
O3	0.2945 (3)	0.46855 (14)	0.70526 (9)	0.0174 (4)	
O4	0.6289 (3)	0.37449 (14)	0.68623 (9)	0.0173 (4)	
H4A	0.6367	0.3789	0.7364	0.026*	
C6	0.3977 (4)	0.41409 (19)	0.57268 (14)	0.0125 (5)	
C7	0.1924 (5)	0.4564 (2)	0.54035 (14)	0.0183 (5)	
H7	0.0767	0.4870	0.5749	0.022*	
C8	0.1538 (5)	0.4548 (2)	0.45852 (14)	0.0201 (6)	
H8	0.0124	0.4839	0.4369	0.024*	
C9	0.3232 (5)	0.41022 (19)	0.40809 (14)	0.0196 (6)	
H9	0.2987	0.4095	0.3518	0.024*	
C10	0.5282 (5)	0.3668 (2)	0.44000 (15)	0.0200 (6)	
H10	0.6438	0.3363	0.4054	0.024*	
C11	0.5651 (5)	0.3678 (2)	0.52216 (14)	0.0162 (5)	
H11	0.7046	0.3369	0.5439	0.019*	
C12	0.4324 (4)	0.42155 (19)	0.66114 (14)	0.0132 (5)	

### Atomic displacement parameters (Å^2^)

**Table:**

	*U*^11^	*U*^22^	*U*^33^	*U*^12^	*U*^13^	*U*^23^
O1	0.0133 (9)	0.0308 (10)	0.0117 (8)	0.0002 (8)	−0.0019 (7)	−0.0037 (7)
O2	0.0085 (9)	0.0172 (8)	0.0135 (8)	0.0008 (7)	−0.0004 (7)	−0.0001 (7)
N1	0.0101 (11)	0.0146 (9)	0.0106 (9)	−0.0001 (8)	−0.0006 (8)	0.0010 (8)
C1	0.0155 (13)	0.0137 (11)	0.0191 (12)	0.0018 (12)	0.0015 (10)	−0.0011 (9)
C2	0.0186 (14)	0.0141 (11)	0.0155 (12)	0.0045 (11)	−0.0008 (10)	0.0015 (9)
C3	0.0139 (13)	0.0181 (12)	0.0127 (12)	0.0029 (11)	0.0020 (10)	0.0016 (10)
C4	0.0108 (12)	0.0128 (11)	0.0094 (11)	0.0004 (10)	0.0011 (9)	−0.0010 (9)
C5	0.0112 (12)	0.0116 (10)	0.0129 (11)	−0.0026 (11)	−0.0005 (10)	0.0022 (9)
O3	0.0169 (10)	0.0200 (9)	0.0152 (8)	0.0022 (8)	0.0045 (7)	0.0013 (7)
O4	0.0186 (10)	0.0231 (9)	0.0101 (8)	0.0053 (8)	−0.0017 (7)	0.0015 (7)
C6	0.0116 (12)	0.0116 (11)	0.0142 (11)	−0.0024 (10)	0.0007 (9)	0.0017 (9)
C7	0.0157 (13)	0.0174 (12)	0.0216 (13)	0.0029 (11)	0.0018 (11)	0.0008 (10)
C8	0.0196 (14)	0.0169 (12)	0.0237 (13)	0.0018 (11)	−0.0068 (12)	0.0044 (11)
C9	0.0272 (15)	0.0168 (12)	0.0147 (12)	−0.0033 (12)	−0.0059 (11)	0.0017 (9)
C10	0.0241 (14)	0.0197 (13)	0.0164 (12)	−0.0006 (12)	0.0005 (11)	−0.0027 (11)
C11	0.0160 (13)	0.0153 (11)	0.0172 (12)	0.0028 (11)	−0.0010 (10)	0.0011 (10)
C12	0.0138 (13)	0.0111 (11)	0.0146 (11)	−0.0032 (10)	0.0026 (10)	0.0027 (9)

### Geometric parameters (Å, º)

**Table:**

O1—C5	1.239 (3)	C4—C5	1.521 (3)
O2—C5	1.266 (3)	O3—C12	1.217 (3)
N1—H1A	0.9100	O4—H4A	0.8400
N1—H1B	0.9100	O4—C12	1.324 (3)
N1—C1	1.498 (3)	C6—C7	1.386 (3)
N1—C4	1.507 (3)	C6—C11	1.391 (3)
C1—H1C	0.9900	C6—C12	1.492 (3)
C1—H1D	0.9900	C7—H7	0.9500
C1—C2	1.517 (3)	C7—C8	1.383 (3)
C2—H2A	0.9900	C8—H8	0.9500
C2—H2B	0.9900	C8—C9	1.389 (4)
C2—C3	1.528 (3)	C9—H9	0.9500
C3—H3A	0.9900	C9—C10	1.386 (4)
C3—H3B	0.9900	C10—H10	0.9500
C3—C4	1.536 (3)	C10—C11	1.387 (3)
C4—H4	1.0000	C11—H11	0.9500
			
H1A—N1—H1B	108.4	C5—C4—C3	112.05 (19)
C1—N1—H1A	110.0	C5—C4—H4	109.6
C1—N1—H1B	110.0	O1—C5—O2	125.8 (2)
C1—N1—C4	108.32 (18)	O1—C5—C4	118.9 (2)
C4—N1—H1A	110.0	O2—C5—C4	115.28 (19)
C4—N1—H1B	110.0	C12—O4—H4A	109.5
N1—C1—H1C	111.1	C7—C6—C11	119.5 (2)
N1—C1—H1D	111.1	C7—C6—C12	118.3 (2)
N1—C1—C2	103.39 (18)	C11—C6—C12	122.2 (2)
H1C—C1—H1D	109.0	C6—C7—H7	119.6
C2—C1—H1C	111.1	C8—C7—C6	120.8 (2)
C2—C1—H1D	111.1	C8—C7—H7	119.6
C1—C2—H2A	111.3	C7—C8—H8	120.2
C1—C2—H2B	111.3	C7—C8—C9	119.6 (3)
C1—C2—C3	102.53 (19)	C9—C8—H8	120.2
H2A—C2—H2B	109.2	C8—C9—H9	120.0
C3—C2—H2A	111.3	C10—C9—C8	120.0 (2)
C3—C2—H2B	111.3	C10—C9—H9	120.0
C2—C3—H3A	110.8	C9—C10—H10	119.9
C2—C3—H3B	110.8	C9—C10—C11	120.3 (2)
C2—C3—C4	104.55 (19)	C11—C10—H10	119.9
H3A—C3—H3B	108.9	C6—C11—H11	120.1
C4—C3—H3A	110.8	C10—C11—C6	119.9 (2)
C4—C3—H3B	110.8	C10—C11—H11	120.1
N1—C4—C3	104.87 (18)	O3—C12—O4	123.7 (2)
N1—C4—H4	109.6	O3—C12—C6	122.7 (2)
N1—C4—C5	110.90 (18)	O4—C12—C6	113.5 (2)
C3—C4—H4	109.6		
			
N1—C1—C2—C3	−39.6 (2)	C7—C6—C11—C10	1.6 (4)
N1—C4—C5—O1	−6.0 (3)	C7—C6—C12—O3	−4.2 (3)
N1—C4—C5—O2	176.36 (18)	C7—C6—C12—O4	177.2 (2)
C1—N1—C4—C3	−4.2 (2)	C7—C8—C9—C10	0.7 (4)
C1—N1—C4—C5	117.0 (2)	C8—C9—C10—C11	−0.1 (4)
C1—C2—C3—C4	37.3 (2)	C9—C10—C11—C6	−1.0 (4)
C2—C3—C4—N1	−20.6 (2)	C11—C6—C7—C8	−1.0 (4)
C2—C3—C4—C5	−141.01 (19)	C11—C6—C12—O3	174.9 (2)
C3—C4—C5—O1	110.8 (2)	C11—C6—C12—O4	−3.7 (3)
C3—C4—C5—O2	−66.8 (3)	C12—C6—C7—C8	178.1 (2)
C4—N1—C1—C2	27.4 (2)	C12—C6—C11—C10	−177.5 (2)
C6—C7—C8—C9	−0.1 (4)		

### Hydrogen-bond geometry (Å, º)

**Table:**

*D*—H···*A*	*D*—H	H···*A*	*D*···*A*	*D*—H···*A*
N1—H1*A*···O2^i^	0.91	1.92	2.751 (3)	151
N1—H1*B*···O1	0.91	2.18	2.679 (3)	114
N1—H1*B*···O1^ii^	0.91	2.08	2.782 (2)	133
C4—H4···O3^iii^	1.00	2.30	3.192 (3)	147
O4—H4*A*···O2^iv^	0.84	1.76	2.595 (2)	173

Symmetry codes: (i) *x*−1, *y*, *z*; (ii) *x*−1/2, −*y*+3/2, −*z*+1; (iii) −*x*+1/2, −*y*+1, *z*−1/2; (iv) −*x*+3/2, −*y*+1, *z*+1/2.
